# Bone Substitutes in Alveolar Ridge Augmentation: A Narrative Literature Review

**DOI:** 10.3390/jfb17040176

**Published:** 2026-04-01

**Authors:** Marija Bubalo, Sanja Dugonjic, Dejan Dubovina, Zdenka Stojanovic, Milka Gardasevic, Jelena Mijatovic, Boban Milovanovic, Milovan Stevic, Milos Stepovic, Dejan Jeremic, Zlata Rajkovic Pavlovic

**Affiliations:** 1 Clinic for Dentistry, Medical Faculty of the Military Medical Academy, University of Defence, 11000 Belgrade, Serbia; bubalo.marija@yahoo.com (M.B.);; 2 Institute of Nuclear Medicine, Medical Faculty of the Military Medical Academy, University of Defence, 11000 Belgrade, Serbia; 3 Department of Dentistry, Faculty of Medicine, University of Pristina in Kosovska Mitrovica, 38220 Kosovska Mitrovica, Serbia; 4 Clinic for Maxillofacial Surgery, Medical Faculty of the Military Medical Academy, University of Defence, 11000 Belgrade, Serbia; 5 Department of Anatomy, Faculty of Medical Sciences, University of Kragujevac, 34000 Kragujevac, Serbia; 6 Department of Dentistry, Faculty of Medical Sciences, University of Kragujevac, 34000 Kragujevac, Serbia; zlatakg@yahoo.com

**Keywords:** alveolar ridge augmentation, bone graft, bone substitute, guided bone regeneration, dental implants, xenograft, alloplast, demineralized dentin matrix, PRF, bone regeneration

## Abstract

Adequate alveolar bone volume is a prerequisite for predictable and long-term success in dental implant therapy. Physiological post-extraction remodeling frequently results in horizontal and vertical ridge deficiencies, which may compromise optimal implant placement. Guided bone regeneration (GBR) has become a cornerstone procedure in implant dentistry, with clinical outcomes largely influenced by the biological and mechanical characteristics of grafting materials. Different bone grafts and their combinations are currently clinically applicable, each exhibiting distinct osteogenic, osteoinductive, and osteoconductive properties, as well as varying resorption profiles and volumetric stability. This narrative review aims to analyze the biological principles of alveolar ridge augmentation, compare the properties of commonly used graft materials, evaluate clinical outcomes, and discuss emerging regenerative strategies. Literature published between 2000 and 2025 was assessed to synthesize current evidence regarding graft integration, bone formation, desorption dynamics, and clinical indications. Autogenous bone remains the gold standard due to its combined osteogenic, osteoinductive, and osteoconductive potential; however, its limitations have driven the development of alternative materials, including allografts, xenografts, alloplastic substitutes, demineralized tooth matrices, platelet concentrates, and customized scaffolds. While no single material is universally ideal, appropriate selection based on defect characteristics and clinical objectives is essential for predictable outcomes. Future research should prioritize long-term comparative trials, biomaterial standardization, and biologically enhanced regenerative approaches.

## 1. Introduction

Alveolar bone volume represents a fundamental requirement for functional, biomechanical, and esthetic success in implant dentistry. Physiological bone remodeling after tooth extraction leads to dimensional ridge changes predominantly by horizontal bone loss and varying degrees of vertical reduction [1]. These structural alterations in bone quantity have a strong impact on implant rehabilitation, proper position, primary stability, load distribution, and long-term peri-implant tissue health [2].

Alveolar bone resorption is a biologically driven process influenced by mechanical unloading, vascular changes, local anatomy, surgical trauma, and systemic factors [1]. The most pronounced reduction typically occurs within the first months after extraction, emphasizing the clinical necessity for ridge preservation and augmentation procedures [3].

The selection of the best option for bone preservation and augmentation remains a clinical challenge. This is partly due to the fact that there are various materials, combinations of which are used, so clinicians often have personal “best choices” because they have been working with the same ones for years [4,5]. An optimal approach would involve tailoring the entire surgical protocol according to the selected bone substitute, ensuring procedural compatibility and maximizing clinical outcomes [6,7].

Guided bone regeneration (GBR) is widely regarded as the most predictable and extensively documented technique for managing localized alveolar defects. Successful regeneration relies on multiple interdependent variables, including defect morphology, membrane stability, angiogenesis, wound closure, and, critically, graft material selection [8]. Bone substitutes serve essential roles in osteoconduction, space maintenance, volumetric stability, and modulation of resorption dynamics [9].

Despite decades of clinical use, consensus regarding the optimal grafting material remains elusive. Each biomaterial category exhibits distinct biological behavior, mechanical properties, integration patterns, and clinical indications [10,11].

While several landmark reviews have provided comprehensive insights into alveolar ridge preservation (ARP) materials and techniques, as well as the biological properties of bone graft substitutes in implant dentistry, important gaps remain in the integration of biomaterial characteristics with clinical decision making across different defect types. In contrast to reviews focused primarily on ARP, this paper encompasses a wider spectrum of augmentation strategies, including combined grafting approaches and diverse biomaterials used in different clinical scenarios. Furthermore, the review emphasizes practical clinical applicability by presenting comparative tables and a decision-oriented framework intended to facilitate material and technique selection in routine practice. In addition, emerging regenerative approaches, such as demineralized tooth matrices and 3D-printed scaffolds, are discussed in relation to their clinical potential and reported outcomes.

This review aims to examine the biological principles underlying alveolar ridge augmentation, compare the characteristics and clinical performance of currently available bone grafts and substitutes, evaluate their limitations, and discuss emerging regenerative technologies while identifying existing gaps in the literature. Through the analysis of relationships between graft type, defect characteristics, and clinical outcomes, it provides an integrative synthesis that connects biomaterials, surgical techniques, and patient-centered results. Based on the currently available published evidence, this research may provide insights suggesting that specific grafts are preferable for achieving positive clinical outcomes across different types of bone defects.

## 2. Materials and Methods

**Figure 1:**
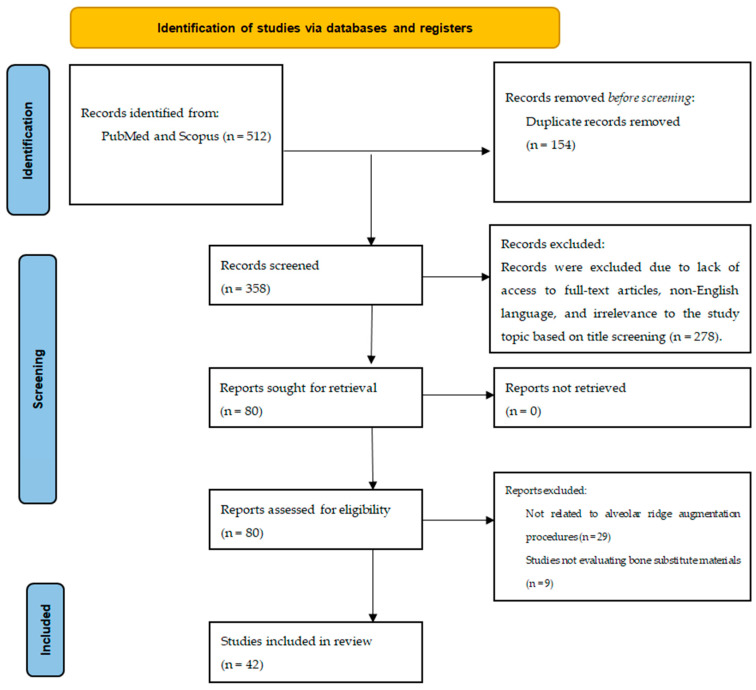
PRISMA flow chart, and process of selection of studies in the research.

This narrative review included peer-reviewed studies retrieved from the PubMed and Scopus databases, without restriction on publication year. The literature search employed combinations of the following keywords: “bone graft,” “bone substitute,” “guided bone regeneration,” “alveolar ridge augmentation,” “dental implants,” “xenograft,” “alloplast,” “demineralized dentin,” and “platelet-rich fibrin.” Boolean operators (AND, OR) were used to combine the terms. An example of the search strategy used in PubMed was: (“alveolar ridge augmentation” OR “guided bone regeneration”) AND (“bone graft” OR “bone substitute” OR “xenograft” OR “alloplast”) AND (“dental implants”). Studies were selected based on relevance to the biological and clinical outcomes of alveolar ridge augmentation and regenerative procedures (Figure 1). The literature selection process was guided by predefined inclusion criteria to ensure the relevance and scientific validity of the reviewed evidence. Eligible publications included human clinical studies, relevant preclinical animal investigations, randomized controlled trials (RCTs), and systematic reviews addressing bone grafting and alveolar ridge augmentation. Studies were considered if they provided data on biological behavior and/or the clinical performance of graft materials. The primary parameters evaluated across the selected literature encompassed osteogenic potential, graft integration and remodeling, resorption patterns, volumetric stability, and implant-related outcomes, including osseointegration and clinical success. Given the substantial heterogeneity observed among studies in terms of biomaterial composition, surgical protocols, defect characteristics, and outcome assessment methods, the findings were synthesized using a qualitative, narrative approach rather than quantitative meta-analysis.

## 3. Bone Grafts and Substitutes

### 3.1. Autogenous Bone

#### 3.1.1. Biological Mechanism

Autogenous bone or autograft is an osteogenic material capable of producing bone even in the absence of local undifferentiated mesenchymal cells. It is a graft composed of the patient’s own bone [12]. Autogenous bone is the only graft material that directly creates bone from transplanted cancellous bone cells [13]. The graft also promotes bone formation through the release of bone morphogenetic proteins (BMPs) during graft incorporation [12,13]. This combination of osteogenic, osteoinductive, and osteoconductive properties makes it the gold standard in augmentation procedures, as it is fully biocompatible without the risk of foreign body reaction [12].

#### 3.1.2. Evidence Summary

Autografts can be harvested from various intraoral sites such as the ramus, tuberosity, symphysis region, and alveolar ridge, as well as extraoral sites including the iliac crest, tibia, or cranium [13,14]. They may be cortical, cancellous, or cortico-cancellous, with cortical grafts demonstrating slower resorption rates [13,14]. Studies have consistently shown predictable bone regeneration; however, significant resorption has also been reported, with up to 55% volume loss within the first 6 months [15].

#### 3.1.3. Clinical Outcomes

Autogenous bone remains the gold standard owing to its intrinsic osteogenic, osteoinductive, and osteoconductive properties [12,13,14]. Viable cellular components and endogenous growth factors directly contribute to new bone formation [15,16]. Clinically, it provides reliable augmentation outcomes, although vertical bone resorption of up to 7% per year may negatively influence implant success rates. The use of additional bone substitutes, such as xenografts, has been shown to improve volumetric stability and increase the percentage of vital bone, particularly in block graft procedures [15,16].

#### 3.1.4. Limitations and Controversies

Despite its advantages, autogenous bone presents several important limitations, including donor site morbidity, limited availability, and variable resorption patterns [12,13,14,15,16,17]. Additional surgical procedures increase patient discomfort and complication risk. Although it remains the gold standard, there is ongoing discussion regarding the necessity of autografts in all clinical situations, especially with the increasing use of alternative biomaterials.

### 3.2. Allografts

#### 3.2.1. Biological Mechanism

A bone allograft is bone tissue transplanted from a donor of the same species but with a different genotype. Allografts primarily exhibit osteoinductive and osteoconductive properties. Osteoinduction is mediated by bioactive molecules, including growth factors that stimulate differentiation of progenitor cells in the recipient site, leading to new bone formation [17]. Demineralized forms, in particular, expose bone morphogenetic proteins (BMPs), enhancing their osteoinductive potential [18,19].

#### 3.2.2. Evidence Summary

Allografts are available in various forms, including frozen, freeze-dried, and demineralized freeze-dried bone [18]. They may be mineralized or demineralized, as well as cortical, cancellous, or cortico-cancellous in structure. Demineralized cortical bone contains higher concentrations of BMPs compared to cancellous bone and is therefore frequently recommended in dental applications [19]. Particle size ranges from 0.25 µm to 2 µm, with smaller particles demonstrating faster resorption rates [17,18,19].

#### 3.2.3. Clinical Outcomes

The major clinical advantage of allografts is the elimination of donor site morbidity and their availability in larger quantities [18]. They are widely used in implantology and alveolar ridge augmentation procedures, particularly in mineralized forms that provide structural support [18,19]. Demineralized allografts enhance biological activity by promoting host cell differentiation, although their integration is generally slower compared to autografts [20].

#### 3.2.4. Limitations and Controversies

Despite their clinical utility, allografts have certain limitations, including slower incorporation and remodeling compared to autogenous bone [20]. Variability in processing methods and graft composition may influence biological performance and clinical outcomes. Additionally, although highly controlled, concerns regarding disease transmission and immunogenic response have historically been discussed, contributing to ongoing debate about their optimal clinical use.

### 3.3. Xenografts

#### 3.3.1. Biological Mechanism

Xenografts are graft materials derived from a different species and primarily exhibit osteoconductive properties. Osteoconduction refers to the ability of the material to serve as a scaffold or matrix that supports the migration, attachment, and proliferation of osteogenic cells [21]. Their micro- and macroporous structure facilitates cellular infiltration and vascularization, enabling new bone formation along the surface of the graft particles [21]. Histological studies have demonstrated the presence of osteoblasts and osteoid tissue on xenogeneic particles, with vital bone gradually forming bridges between particles over time [22].

#### 3.3.2. Evidence Summary

Xenografts are most commonly of bovine origin, although porcine and equine sources are also used [23,24]. They are available in various physical forms, differing in granule size, shape, porosity, and crystallinity [24]. These materials can be classified as resorbable or non-resorbable, as well as dense or porous structures, depending on their processing and composition [23,24]. Their structural similarity to human bone has been shown to support predictable osteoconductive behavior in preclinical and clinical studies.

#### 3.3.3. Clinical Outcomes

Clinically, xenografts are widely used in alveolar ridge augmentation and sinus lift procedures due to their ability to maintain volume and provide long-term structural stability [23,24]. Their slow resorption rate contributes to space maintenance, which is particularly beneficial in preventing collapse of the augmented site. Over time, newly formed bone integrates with the graft particles, increasing the amount of vital bone within the augmented area [22,25].

#### 3.3.4. Limitations and Controversies

Despite their advantages, xenografts present certain limitations. Their slow resorption may delay complete physiological remodeling and replacement by native bone [25]. Residual graft particles may persist for extended periods, which can influence bone quality and remodeling dynamics. Additionally, variability in physicochemical properties between different xenogeneic materials may affect clinical outcomes, leading to ongoing discussion regarding their optimal indication and long-term behavior.

### 3.4. Alloplastic Materials

#### 3.4.1. Biological Mechanism

Alloplastic materials are synthetic graft substitutes that primarily exhibit osteoconductive properties by providing a scaffold for bone formation. The most commonly used materials include β-tricalcium phosphate (β-TCP), which is resorbable, and hydroxyapatite (HA), which is non-resorbable or very slowly soluble [26]. These materials support bone regeneration through their physicochemical characteristics, including porosity and surface structure, which facilitate cellular attachment and tissue ingrowth. However, due to the absence of intrinsic biological components, they lack osteoinductive potential unless combined with biologically active agents [27,28,29].

#### 3.4.2. Evidence Summary

Synthetic grafts offer controlled and reproducible physicochemical properties, allowing modulation of resorption rates and mechanical stability. β-TCP demonstrates faster resorption and replacement by newly formed bone, whereas HA provides prolonged structural support due to its slow degradation [28]. These materials are often used in combination to achieve a balance between stability and resorption [26,27]. Numerous studies and meta-analyses have confirmed the importance of bone substitutes in augmentation procedures, with outcomes influenced by the type of material used [16,18,23,24,28].

#### 3.4.3. Clinical Outcomes

Clinically, alloplastic materials are widely used due to their availability, absence of donor site morbidity, and lack of antigenic reaction [27]. They are frequently applied in combination with barrier membranes, which significantly improve clinical outcomes in alveolar ridge augmentation [28]. Histological studies have demonstrated new bone formation at grafted sites, with an average residual presence of graft particles of approximately 25% [29]. Their use allows for successful ridge augmentation and, in certain cases, enables implant placement within approximately 6 months [29].

#### 3.4.4. Limitations and Controversies

Despite their advantages, alloplastic materials have certain limitations. The lack of osteoinductive properties may result in less favorable outcomes compared to autogenous grafts, particularly in large or complex defects [28]. Clinical results may depend on adjunctive techniques, such as the use of membranes or biologics. Additionally, incomplete resorption and persistence of graft particles may influence long-term remodeling dynamics. These factors contribute to ongoing debate regarding their optimal role and indications in regenerative procedures.

### 3.5. Demineralized Tooth Matrix

#### 3.5.1. Biological Mechanism

In recent years, teeth have emerged as a potential autogenous graft material due to their favorable biological composition. The mineral component of dentin consists of multiple calcium phosphates, including hydroxyapatite, tricalcium phosphate, octacalcium phosphate, amorphous calcium phosphate, and dicalcium phosphate dihydrate [30], which contribute to its ability to support bone remodeling. Following demineralization, the matrix releases bioactive molecules such as bone morphogenetic proteins (BMPs), which induce mesenchymal cell differentiation into osteoblasts [31]. These osteoblasts produce a mineralized matrix that forms new bone, eventually differentiating into osteocytes that integrate within the newly formed tissue [32]. Mineralization processes are further supported by enzymes such as alkaline phosphatase and phosphophorin, contributing to both dentin and newly formed bone mineralization [33]. New bone gradually forms around the demineralized dentin scaffold, which is ultimately replaced by native bone tissue [31,32,33,34].

#### 3.5.2. Evidence Summary

Various chemical and physical processing techniques are used to prepare demineralized dentin matrix as a bone substitute. Histological studies have demonstrated active bone formation around dentin particles, including the presence of osteoblasts, osteoid tissue, and progressive remodeling patterns [31,32,33,34]. These findings support its classification as a biologically active material with both osteoinductive and osteoconductive properties [35].

#### 3.5.3. Clinical Outcomes

Clinically, demineralized tooth matrix has shown favorable integration and remodeling characteristics, allowing for successful alveolar ridge augmentation. Studies indicate that implant placement may be possible within 4–6 months following augmentation procedures [35]. Its autogenous origin ensures high biocompatibility and eliminates the risk of immune reaction, making it an attractive alternative to conventional grafting materials.

#### 3.5.4. Limitations and Controversies

Despite promising results, the clinical use of demineralized tooth matrix is limited by the availability of suitable teeth, as not all patients have an extractable tooth that can be utilized as graft material [35]. Additionally, variability in preparation protocols and material properties may influence clinical outcomes. While early results are encouraging, further standardized clinical studies are required to fully establish its long-term predictability and broader clinical applicability.

### 3.6. Platelet Concentrates

#### 3.6.1. Biological Mechanism

Platelet concentrates have emerged as biologically active adjuncts in regenerative dentistry due to their ability to enhance wound healing and modulate tissue regeneration. The most commonly used preparations include platelet-rich plasma (PRP) and platelet-rich fibrin (PRF), both derived from autologous blood through centrifugation protocols [36]. Unlike conventional grafting materials, platelet concentrates do not serve as structural scaffolds but function as biological mediators that influence cellular behavior and tissue repair [37]. Their regenerative potential is attributed to a high concentration of growth factors, including platelet-derived growth factor (PDGF), transforming growth factor beta (TGF-β), vascular endothelial growth factor (VEGF), and insulin-like growth factor (IGF), which play key roles in angiogenesis, fibroblast proliferation, collagen synthesis, and osteoblastic differentiation [38]. Enhanced vascularization is essential for successful bone regeneration, facilitating nutrient delivery, cellular migration, and graft integration [38].

#### 3.6.2. Evidence Summary

PRP is considered a first-generation platelet concentrate characterized by the rapid release of growth factors, whereas PRF represents a second-generation preparation with a more sustained release profile due to its fibrin matrix structure [36]. The fibrin network in PRF acts as a reservoir for cytokines and growth factors, prolonging their biological activity. Additionally, PRF offers practical advantages, including ease of preparation and the absence of anticoagulants or biochemical additives [36]. Numerous studies have demonstrated that platelet concentrates can positively influence early phases of healing and tissue regeneration when used in regenerative procedures [36,37,38,39,40,41].

#### 3.6.3. Clinical Outcomes

In alveolar ridge augmentation, platelet concentrates are most commonly used in combination with bone grafts or substitutes [39]. Their adjunctive use has been associated with improved early healing, enhanced soft tissue maturation, reduced postoperative discomfort, and potentially accelerated bone regeneration [40]. Furthermore, they may contribute to improved graft stability by promoting cellular adhesion and early vascular infiltration. Although they do not provide structural support, their role in optimizing the regenerative environment is clinically significant [36,37,38,39,40,41,42].

#### 3.6.4. Limitations and Controversies

Despite their benefits, platelet concentrates present several limitations. Their regenerative potential is highly dependent on patient-related factors, including systemic health, platelet count, and variability in preparation protocols [41]. The lack of mechanical strength prevents their use as standalone graft materials in defects requiring structural support. Additionally, differences in preparation techniques complicate standardization and comparison of clinical outcomes across studies [41]. Consequently, their primary role remains adjunctive rather than substitutive in regenerative procedures.

### 3.7. 3D-Printed Scaffolds

#### 3.7.1. Biological Mechanism

Advancements in digital technologies and biomaterial engineering have introduced three-dimensional (3D) printing as an innovative approach in bone regeneration and alveolar ridge augmentation. 3D-printed scaffolds function primarily as osteoconductive structures, providing a customizable framework that supports cellular migration, vascular infiltration, and new bone formation. Their biological performance is largely determined by structural and physicochemical characteristics, including pore size, porosity, and interconnectivity, which facilitate nutrient diffusion and angiogenesis [43,44]. Adequate porosity promotes osteogenesis through the formation of a functional microvascular network. Additionally, mechanical properties can be engineered to mimic native bone, allowing structural support and gradual load transfer during remodeling [45].

#### 3.7.2. Evidence Summary

A wide range of biomaterials is used in scaffold fabrication, including bioceramics (such as hydroxyapatite and β-tricalcium phosphate), polymers, and composite materials [46]. Bioceramic scaffolds provide osteoconductive properties, while polymeric components contribute to flexibility and controlled degradation. Composite scaffolds aim to combine mechanical strength with biological functionality. One of the key advantages of 3D printing is the ability to produce patient-specific constructs that precisely match defect morphology, improving adaptation and stability [43]. Furthermore, scaffolds can be functionalized with growth factors, bioactive molecules, or stem cells to enhance osteoinductive potential [47].

#### 3.7.3. Clinical Outcomes

In alveolar ridge augmentation, 3D-printed scaffolds offer potential benefits, particularly in complex or irregular defects where conventional graft adaptation may be limited [46,48]. Customized design improves defect conformity, reduces dead space, and enhances graft stability, potentially leading to improved volumetric outcomes. Integration with digital workflows also enables preoperative planning and increases surgical precision and predictability [48]. Although early clinical applications show promising results, most available evidence is derived from preclinical studies and limited clinical trials [46].

#### 3.7.4. Limitations and Controversies

Despite their potential, 3D-printed scaffolds face several limitations. Clinical evidence remains limited, and long-term outcomes regarding scaffold resorption, mechanical stability, and implant success are not yet fully established [46,47,48]. High manufacturing costs, technical complexity, and regulatory challenges currently restrict widespread clinical implementation [47]. Additionally, variability in material composition and fabrication techniques may influence biological performance. Therefore, further research and clinical validation are necessary to confirm their long-term predictability and clinical superiority.

### 3.8. Surgical Considerations

Immediate implant placement is now routinely associated with regenerative procedures. Contemporary protocols typically incorporate guided bone regeneration, including the use of membranes and bone substitutes, as well as soft tissue grafting when indicated. The success of immediate implantation depends on several factors [49]. Provided that the implant is positioned in an adequate three-dimensional location and achieves satisfactory primary stability, a gap is intentionally created between the buccal implant surface and the buccal lamella [4].

The management of this peri-implant gap is influenced by multiple parameters, including gap size, the thickness and integrity of the buccal lamella (e.g., presence of fenestrations), and the gingival biotype. Biologically, this space allows formation of a blood clot that organizes into a provisional connective tissue matrix, subsequently supporting immature woven bone and later mature lamellar bone [50]. Clinically, gaps up to 2 mm may heal spontaneously, although a bone substitute may still be applied. For gaps exceeding 2 mm, bone substitutes are typically placed either within the gap or extended over the implant and buccal lamella to enhance contour stability [51]. Although some studies suggest that grafting may be unnecessary in small gaps, the majority of evidence indicates that bone substitutes help increase healing, reduce post-extraction resorption, preserve ridge architecture, decrease recession risk, and improve bone-to-implant contact. Therefore, augmentation of both the gap and adjacent buccal contour is frequently recommended to optimize hard and soft tissue stability [52].

The choice of graft placement technique depends largely on whether immediate loading is planned [26]. In immediate implantation without loading, the implant is placed first, followed by grafting and membrane application [4,49]. In immediate implantation with temporization, several approaches may be used, including graft placement prior to implant insertion, graft placement following preparation of the implant bed, or graft application after provisional restoration [9,53]. These variations reflect differences in surgical preference and defect morphology, while the biological objective remains consistent: stabilization of the regenerative environment and preservation of peri-implant tissues [53]. Defect morphology, graft stability, gap management, and implant positioning critically influence regenerative success. Excessive micromotion may impair ossification and favor fibrous tissue formation [54].

### 3.9. Soft Tissue Considerations

Successful implant therapy requires not only sufficient bone volume but also favorable peri-implant soft tissue conditions [55]. The long-term stability, biological health, and esthetic integration of implants depend on the harmonious interaction between hard and soft tissues [56]. In particular, adequate soft tissue thickness and the presence of keratinized mucosa play a critical role in maintaining peri-implant tissue health, facilitating effective oral hygiene, and ensuring optimal esthetic outcomes [55,56,57]. Beyond tissue thickness, additional clinical parameters such as the width of the keratinized mucosa and the mobility of the marginal tissues are equally important determinants of peri-implant stability [57].

From a clinical standpoint, soft tissue augmentation procedures, often performed in conjunction with osseous augmentation, pursue two principal objectives: increasing the width of the attached and keratinized peri-implant mucosa and enhancing overall soft tissue thickness [58]. Achieving these goals contributes to improved tissue resistance, reduced risk of recession, enhanced patient comfort during hygiene procedures, and superior esthetic contouring [56,57,58,59,60].

Flap design represents a key surgical factor influencing soft tissue outcomes. Careful planning is essential to prevent reduction of the attached gingiva, particularly in procedures requiring flap elevation [60]. In cases where full-thickness flaps are necessary, periosteal-releasing incisions at the flap base may be employed to enable tension-free adaptation, as described in techniques such as the “sausage technique.” Whenever feasible, vertical-releasing incisions are minimized or avoided to preserve vascularization and optimize soft tissue appearance, while horizontal extensions may be preferred [61,62].

Depending on defect characteristics and clinical objectives, various soft tissue augmentation techniques may be considered, including free gingival grafts, connective tissue grafts, pedicled roll flaps, or the adjunctive use of barrier membranes integral to guided bone regeneration protocols [56,58]. Soft tissue thickness and keratinized mucosa width significantly influence peri-implant stability, esthetics, and biological sealing [55,56,57,58,59,60,61]. The comparative characteristics of bone grafts and substitutes are presented in Table 1.

## 4. Discussion

Dental implant success is fundamentally dependent on the quantity and quality of alveolar bone, which determine primary stability, functional load distribution, and long-term biomechanical performance. Post-extraction dimensional changes represent a major biological limitation, with reported reductions ranging from 29–63% horizontally and 11–22% vertically within 6 months [62]. Bone remodelation and possible defects complicate implant positioning and prosthetic planning, thereby emphasizing the clinical importance of bone preservation and augmentation procedures [63,64,65]. The coordinated interaction between hard and soft tissues remains essential, as preservation of peri-implant tissue integrity directly influences treatment predictability.

Bone substitutes function as implantation or transplantation materials of human, animal, or synthetic origin designed to restore lost volume while supporting regeneration through osteoconductive, osteoinductive, and occasionally osteogenic mechanisms [66,67,68]. Clinical outcomes are governed by the dynamic interplay between host bone and graft material, particularly processes of vascularization, incorporation, remodeling, and replacement.

Autogenous bone continues to represent the biological gold standard due to its combined osteogenic, osteoinductive, and osteoconductive properties. The presence of viable cells and growth factors enables rapid regeneration; however, donor site morbidity, limited availability, and unpredictable resorption remain significant limitations [15]. Reported resorption rates approaching 55% within 6 months highlight the challenge of maintaining augmented volume, especially in vertical and block grafting procedures [15,16]. Consequently, composite grafting strategies have gained prominence, combining autogenous bone with slowly resorbing substitutes to enhance volumetric stability.

Allografts provide an effective alternative by eliminating donor site complications while preserving osteoconductive and osteoinductive potential, particularly in demineralized forms [17]. Demineralized bone matrix (DBM) promotes new bone formation through the release of bone morphogenetic proteins, although integration kinetics and clinical performance may vary depending on processing techniques and particle characteristics [17,18]. This suggests that their clinical effectiveness largely depends on appropriate material selection and indication, particularly when balancing the need for biological stimulation with adequate structural support in regenerative procedures [17,18,19].

Xenografts are widely utilized because of their favorable space-maintaining capacity and slow resorption profile. They are widely favored for their ability to maintain space and ensure volumetric stability over time, which is critical in procedures such as sinus lift and ridge preservation [24,25]. Their porous architecture supports vascular invasion and cellular migration, facilitating new bone formation [25]. Nevertheless, prolonged persistence of residual particles has raised questions regarding long-term remodeling dynamics and physiological replacement [25].

Synthetic (alloplastic) materials offer advantages including unlimited availability, the absence of disease transmission risk, and controlled physicochemical properties. Their regenerative potential is predominantly osteoconductive, making defect morphology, particle size, porosity, and dissolution kinetics critical determinants of clinical performance [69]. Larger particles generally contribute to improved volume maintenance, whereas smaller particles exhibit accelerated resorption.

Across all biomaterials, an inverse relationship between resorption rate and volume stability remains a central biological consideration [70]. Rapidly resorbing materials may compromise structural support, while slowly resorbing scaffolds may delay physiological remodeling. Achieving an optimal balance between persistence and replacement continues to represent a primary objective in biomaterial development.

Demineralized tooth-derived materials have demonstrated promising osteoinductive and osteoconductive properties, presenting a biologically favorable autogenous alternative [39,40]. Autologous dentin grafts (ADGs), introduced into clinical practice following technological advances since 2008, have shown encouraging regenerative potential [71]. Experimental studies have confirmed their osteoinductive capacity, integration, and controlled resorption [72], while radiographic assessments using CBCT have supported successful incorporation [73]. Comparative clinical investigations have reported favorable bone regeneration outcomes using demineralized autologous dentin grafts relative to xenograft–autogenous combinations [74]. Additionally, ADG offers practical clinical advantages, including reduced surgical time, elimination of donor site morbidity, and lower procedural costs [74].

Biological enhancement strategies further complement graft performance. Platelet concentrates, particularly PRF, have demonstrated beneficial effects on angiogenesis and early healing dynamics. Although PRF lacks structural properties, adjunctive application may improve regenerative outcomes. Clinical studies have shown comparable performance when PRF is combined with xenografts or autogenous mixtures [75]. Recent investigations involving Titanium-PRF (T-PRF) combined with nanocrystalline hydroxyapatite or demineralized bone matrix reported significant bone fill and ridge width gains within 14–16 weeks [76].

Hyaluronic acid (HYA) has emerged as a biologically active adjunct due to its osteoinductive, anti-inflammatory, and antimicrobial properties. Evidence suggests potential benefits in socket preservation and early bone formation [77,78]. However, systematic analyses reveal inconsistent outcomes, with some studies demonstrating significant improvements while others report limited effects on bone or soft tissue preservation [79]. Certain clinical findings have even indicated increased crestal bone loss when HYA or PRF were used [80]. These discrepancies highlight the need for further controlled investigations.

Technological innovations, including 3D-printed scaffolds, represent a shift toward defect-specific regenerative solutions. Customized architecture and optimized porosity offer theoretical advantages, although long-term clinical validation remains limited [45,46,47,48].

Contemporary regenerative protocols increasingly favor composite grafting approaches. Xenograft materials are frequently combined with autogenous bone and biological modifiers to balance osteogenic potential with volumetric stability. Such combinations have demonstrated predictable outcomes in guided bone regeneration (GBR), sinus augmentation, and ridge reconstruction [81]. Clinical evidence suggests that graft composition ratios influence regenerative outcomes. A pilot study comparing various mixtures reported the highest new bone formation in a 1:1 xenograft–autogenous bone combination (65.8%) [82], consistent with findings from recent systematic reviews [83].

Nanocrystalline hydroxyapatite (HA), a calcium phosphate ceramic closely resembling the mineral phase of bone, has demonstrated favorable biocompatibility and osteoconductive properties. Clinical studies indicate positive effects on defect fill, periodontal regeneration, and long-term stability in atrophic maxilla cases [84,85]. Comparative investigations evaluating HA and biphasic calcium phosphate have also reported satisfactory preservation of ridge dimensions [86,87].

Demineralized bone matrix (DMBM), particularly when combined with PRF, has shown promising regenerative potential, although its routine clinical use remains limited [76,88]. From a surgical standpoint, successful regeneration depends not only on biomaterial selection but also on defect management, graft stabilization, micromotion control, and effective soft tissue isolation [29]. Proper membrane application, aseptic technique, and tension-free primary closure remain critical determinants of clinical success.

Soft tissue conditions represent an equally important factor in implant longevity. Adequate mucosal thickness and keratinized tissue width contribute to biological sealing, peri-implant stability, and esthetic outcomes [59,60,61]. The uses of different materials in augmentation protocols and their success are showed in Table 2, while practical clinical guidance for augmentation procedures is shown in Table 3. The level of evidence (LoE) was assigned based on the highest available level of evidence from the cited literature, following established evidence hierarchies, including systematic reviews, randomized controlled trials, and observational clinical studies.

To date, no single graft material has proven universally ideal for alveolar ridge augmentation, highlighting the need for context-specific clinical guidance. Based on current evidence, material selection should consider defect morphology, patient factors, and procedural goals. For horizontal defects, commonly preferred strategies include xenografts or allografts combined with resorbable collagen membranes, occasionally augmented with platelet-rich fibrin (PRF) to enhance soft tissue healing and angiogenesis. For vertical defects, combinations of autografts with alloplasts or xenografts, stabilized by barrier membranes, are often recommended to maximize volumetric gain and structural support. Emerging 3D-printed scaffolds offer patient-specific solutions for complex defects, although cost and long-term evidence remain limiting factors [48]. A comparative overview of these approaches, including practical considerations such as predictability, handling, and cost-effectiveness, is presented in Table 4 to aid clinicians in evidence-based decision making.

From a clinical perspective, cost-effectiveness represents an important factor when selecting grafting materials for alveolar ridge augmentation. Traditional xenografts remain widely used due to their broad availability, relatively lower cost, and well-documented clinical performance in ridge preservation and augmentation procedures [89,90]. In contrast, emerging 3D-printed scaffolds enable highly personalized reconstruction through digital planning and additive manufacturing, which may improve anatomical adaptation and surgical precision [48]. However, these technologies currently involve higher production costs, specialized equipment, and limited long-term clinical evidence, which restrict their widespread routine use [48]. Consequently, while xenografts remain a cost-effective and predictable option for many clinical scenarios, 3D-printed scaffolds may be particularly advantageous in complex defects where patient-specific design can justify the additional cost [48,89,90].

Despite considerable variability in study designs, implant survival rates above 90% are frequently reported in augmented sites, supporting the overall clinical feasibility of bone-substitute-based regenerative approaches. However, meaningful interpretation of these outcomes requires caution. Differences in biomaterial characteristics, surgical protocols, defect morphology, and evaluation methods substantially limit direct comparisons and complicate evidence synthesis. The relatively small number of long-term randomized controlled trials further constrains the ability to draw definitive conclusions regarding the superiority of specific grafting materials.

Important limitations within the existing literature should therefore be recognized. As a narrative (non-systematic) review, this study does not include a formal systematic search strategy or quantitative synthesis, which may introduce selection bias and limit reproducibility. Long-term data addressing volumetric stability, remodeling dynamics, and sustained implant performance remain insufficient. Biomaterial heterogeneity, including variations in composition, particle size, porosity, crystallinity, and resorption behavior, introduces additional complexity when attempting cross-study interpretation. Outcome assessment lacks uniformity, as studies rely on diverse radiographic, histological, and clinical parameters, often applying inconsistent definitions of success. Methodological variability is further amplified by differences in surgical techniques, defect configurations, patient-related factors, and follow-up durations. Collectively, these factors represent a significant barrier to generating high-level evidence and establishing universally accepted clinical guidelines. Future investigations should prioritize methodological standardization, harmonized outcome measures, and rigorously designed long-term comparative trials, with particular emphasis on strategies that balance volumetric stability with physiological bone remodeling.

## 5. Conclusions

Alveolar ridge augmentation represents a biologically complex and clinically demanding procedure in contemporary implant dentistry. Although a wide range of grafting materials is currently available, no single bone substitute fulfills all regenerative requirements. Each material exhibits distinct biological behavior, mechanical properties, and resorption dynamics, necessitating careful selection based on defect characteristics, clinical objectives, and patient-specific factors. Evidence-based clinical “algorithms” can guide material selection, for example, xenografts or allografts with resorbable membranes for horizontal defects, and autograft-based combinations for vertical defects, facilitating predictable outcomes in routine practice. Autogenous bone remains the gold standard due to its superior biological potential; however, limitations such as donor site morbidity and resorption have driven the expanded use of alternative substitutes, including allografts, xenografts, synthetic materials, biologically enhanced grafts, and emerging technologies such as 3D-printed scaffolds. Cost-effectiveness, handling, and long-term volumetric stability are important considerations in choosing the optimal grafting strategy. Current evidence supports the clinical feasibility and predictability of these approaches when applied within appropriate regenerative protocols, particularly guided bone regeneration principles and soft tissue management strategies. Nevertheless, unresolved challenges persist, highlighting the need for standardized research methodologies, long-term clinical outcomes, and the development of biologically enhanced substitutes aimed at improving both regenerative efficiency and tissue stability.

## Figures and Tables

**Table 1:** Comparative characteristics of bone grafts and substitutes.

Graft Type	Origin	Biological Properties	Resorption Profile	Advantages	Limitations	Clinical Indications
Autograft	Patient	Osteogenic + Osteoinductive + Osteoconductive	Moderate to high (cortical slower)	Gold standard; highest regenerative potential	Donor site morbidity; limited volume	Small to large defects; block grafting
Allograft	Human donor	Osteoinductive + Osteoconductive	Variable	No donor site surgery; abundant supply	Slower integration; processing variability	Moderate defects
Xenograft	Animal origin	Osteoconductive	Slow	Excellent volume maintenance	No osteogenic cells; slow remodeling	Ridge augmentation; sinus lift
Alloplast	Synthetic	Osteoconductive	Slow to moderate	No disease transmission; customizable	Limited osteoinduction	Small/contained defects
Demineralized Tooth Matrix	Patient tooth	Osteoinductive + Osteoconductive	Moderate	Autogenous; BMP release	Limited availability	Extraction sites
PRF/PRP	Patient blood	Growth-factor-enhanced	Not applicable	Accelerated healing; angiogenesis	Adjunct only	Combined regenerative procedures
3D-Printed Scaffold	Synthetic/customized	Osteoconductive ± Osteoinductive	Variable	Precise defect adaptation	Cost; limited availability	Complex defects

**Table 2:** The uses of different materials in augmentation protocols and their success.

Study/Year	Graft Composition & Ratio	PRF Use	HA Use	DMBM/Demineralized Matrix	Key Outcomes
Kamat et al., 2025 [82]	Xenograft + Autogenous 1:1	Yes (i-PRF)	/	No	Highest new bone formation (65.8%) with 1:1 + i-PRF in GBR
	Xenograft + Autogenous 2:1	Yes (i-PRF)	/	No	Lower new bone vs. 1:1
	Xenograft only	Yes (i-PRF)	/	No	Lowest new bone
Patil et al., 2024 [76]	Nano-HA + T-PRF	Yes (T-PRF)	Yes (nano-HA)	/	Ridge preservation increase, significant bone fill
	DMBM + T-PRF	Yes (T-PRF)	/	Yes (DMBM)	Comparable bone fill increase vs. HA group
Caramês J.M.M., et al., 2022 [88]	DBBM (xenograft) + PRF	Yes (PRF)	/	No	Effective horizontal bone gain in atrophic maxilla
Shaikh M.S. et al., 2021 [86]	PRF vs. DMBM	Yes (PRF)	/	Yes (DMBM)	PRF showed faster healing than DMBM
PRF + HA (periodontal)	HA + PRF	Yes (PRF)	Yes (HA)	No	Increased defect fill in periodontal defects
Animal studies (DDM)	DDM + PRF (no clinical human ratio)	Yes (PRF in animals)	/	Yes (DDM)	Mixed results; no significant added new bone vs. DDM alone in rabbit calvaria
DDM autograft case	Autogenous DDM only	Yes (PRP used clinically)	/	Yes (DDM)	Long-term successful sinus augmentation

**Table 3:** Practical clinical guidance for augmentation procedures.

Procedure	Option A (Preferred)	Option B (Alternative)	Option C (When A/B Not Possible)	Clinical Consideration	LoE
GBR around implants	Autogenous + DBBM (1:1) ± PRF	DBBM alone + PRF	Alloplast + PRF	Use B if autogenous bone not available	II–III
Sinus lift	DBBM alone	DBBM + 20–30% autogenous bone	Alloplast (β-TCP or HA)	C used when xenografts contraindicated	I–II
Horizontal ridge augmentation	Autogenous + DBBM (1:1)	Allograft + xenograft	Xenograft alone	B/C when donor site surgery avoided	II–III
Vertical ridge augmentation	Autogenous block + DBBM (30–50%)	Autogenous particulate + xenograft	Allograft + xenograft	Autogenous component needed for vertical gain	III–IV

**Table 4:** Clinical guidance for graft selection.

Defect Type	Recommended Graft Strategy	Notes/Advantages	Cost Considerations
Horizontal	Xenograft or Allograft + Resorbable Collagen Membrane ± PRF	Good volumetric stability, predictable outcomes, minimally invasive	Moderate, widely available
Vertical	Autograft + Alloplast/Xenograft + Membrane	Maximizes bone height gain, structural support	Higher, donor site morbidity if autograft used
Complex/Irregular	3D-Printed Scaffold ± Growth Factors	Customizable, potentially superior fit and regeneration	High, limited long-term clinical data

## Data Availability

No new data were created or analyzed in this study. Data sharing is not applicable to this article.
